# Quantifying force transmission through fibroblasts: changes of traction forces under external shearing

**DOI:** 10.1007/s00249-021-01576-8

**Published:** 2021-10-28

**Authors:** Steven Huth, Johannes W. Blumberg, Dimitri Probst, Jan Lammerding, Ulrich S. Schwarz, Christine Selhuber-Unkel

**Affiliations:** 1 Institute of Materials Science, Biocompatible Nanomaterials, Kiel University, Kaiserstr. 2, 24143 Kiel, Germany; 2 Institute for Theoretical Physics and Bioquant-Center for Quantitative Biology, Heidelberg University, Philosophenweg 19, 69120 Heidelberg, Germany; 3 Weill Institute for Cell and Molecular Biology and Meinig School of Biomedical Engineering, Cornell University, 235 Weill Hall, Ithaca, NY 14853 USA; 4 Institute for Molecular Systems Engineering (IMSE), Heidelberg University, INF 253, 69120 Heidelberg, Germany

**Keywords:** Cell adhesion, Mechanobiology, Traction force microscopy, Micromanipulation

## Abstract

Mammalian cells have evolved complex mechanical connections to their microenvironment, including focal adhesion clusters that physically connect the cytoskeleton and the extracellular matrix. This mechanical link is also part of the cellular machinery to transduce, sense and respond to external forces. Although methods to measure cell attachment and cellular traction forces are well established, these are not capable of quantifying force transmission through the cell body to adhesion sites. We here present a novel approach to quantify intracellular force transmission by combining microneedle shearing at the apical cell surface with traction force microscopy at the basal cell surface. The change of traction forces exerted by fibroblasts to underlying polyacrylamide substrates as a response to a known shear force exerted with a calibrated microneedle reveals that cells redistribute forces dynamically under external shearing and during sequential rupture of their adhesion sites. Our quantitative results demonstrate a transition from dipolar to monopolar traction patterns, an inhomogeneous distribution of the external shear force to the adhesion sites as well as dynamical changes in force loading prior to and after the rupture of single adhesion sites. Our strategy of combining traction force microscopy with external force application opens new perspectives for future studies of force transmission and mechanotransduction in cells.

**Supplementary Information:**

The online version contains supplementary material available at 10.1007/s00249-021-01576-8.

## Introduction

Cells exert forces to interact with their surroundings and have the striking ability to react to externally applied forces and mechanical cues by a process called mechanotransduction (Jaalouk and Lammerding 2009; Petridou et al. 2017; Roca-Cusachs et al. 2017). Cellular reactions to external mechanical cues play a crucial role in cellular processes such as stem cell differentiation, adhesion, migration, and proliferation (Paluch 2015; Lv 2015; Engler et al. 2006; Cui 2015; Brugués 2014). Furthermore, focal adhesion clusters grow in response to external shearing (Riveline 2001; Paul et al. 2008) which might help cells to withstand shear forces, e.g., forces exerted by the blood flow on endothelial cells (Davies 1995; Perrault 2015).

Traction force microscopy (TFM) has become an established tool to quantify forces exerted by single cells or cell layers to the underlying substrate, which has deepened our understanding of cell migration, mechanotransduction and cell–matrix interaction (Lo et al. 2000; Schwarz and Soiné 2015; Style 2014; Kronenberg 2017; Sabass et al. 2008; Balaban 2001; Vishwakarma 2018; Hino et al. 2020). However, current traction force microscopy models assume an equilibrium of a cell’s traction forces, whereas in nature cells experience a variety of externally applied forces, for instance from blood flow, muscle contraction, movement of other cells, or wound opening. Force transmission is particularly important in tissue formation and adaption (Ng et al. 2014) as well as in collective cell migration, where many cells interact with each other and mechanically strong cells become leader cells (Das 2015; Vishwakarma 2018). Thus, to understand force transmission by cells more completely, it is crucial to study traction forces under external forces.

Techniques to exert mechanical stimuli to cells include atomic force microscopy (AFM), which can be employed to measure forces necessary to rupture cellular adhesions (Kadem 2016; Selhuber-Unkel 2010) or forces exerted by cells (Huth et al. 2017; Brunner 2006), hydrodynamic shear stress (Davies 1995; Perrault 2015; Hanke et al. 2019), optical or magnetic tweezers (Rief et al. 1997; Neuman and Nagy 2008; Jiang et al. 2003; Roca-Cusachs et al. 2017), microneedle assays (Fedorchak and Lammerding 2016; Riveline 2001; Paul et al. 2008) and optical stretchers (Chan 2015; Micoulet et al. 2005). Despite the fact that such a large variety of physical cell manipulation techniques has been established and cellular forces exerted to surfaces can be measured via TFM or elastic resonator interference stress microscopy (Kronenberg 2017), a quantification of cellular force adaptation as a response to well-defined mechanical stimuli applied to cells has not yet been realized.

Here, we present a new tool that combines TFM with externally applied mechanical stimulation by microneedle shearing. This setting allows to quantify cellular force transmission by measuring how cells distribute an external well-defined shear force to their adhesion sites. The spring constant of the microneedle is calibrated and thus the shear force exerted by the needle is known. We advanced current TFM procedures to create a novel procedure that analyzes traction forces in the presence of an external force monopole. This new force transmission assay is a versatile technique that is complementary to existing methods, as it can also be combined with other techniques such as AFM to broaden our understanding of the interplay of cellular biomechanics and adhesion.

## Results and discussion

**Fig. 1:**
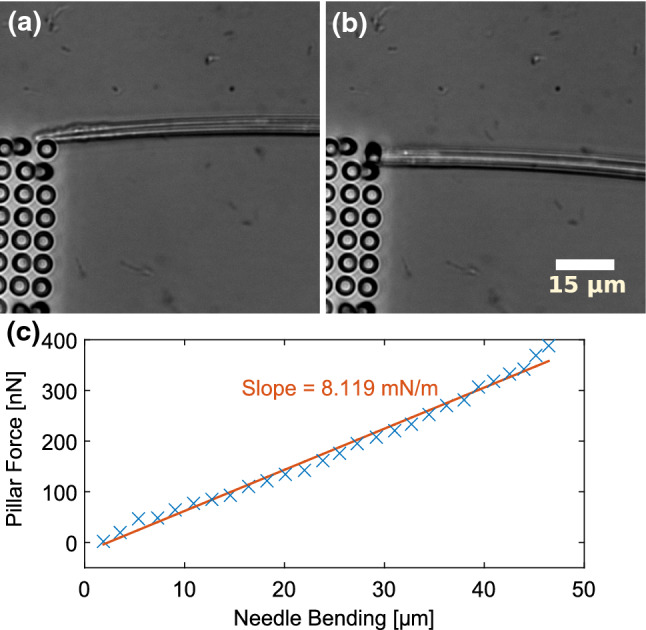
**A**, **B** show exemplary phase contrast images of a microneedle shearing a PDMS pillar. The needle moves downwards and bends the pillar. The pillar, on the other hand, exerts a force to the needle, which results in a bending of the needle. The force acting between needle and pillar is calculated from the bending of the PDMS pillar. **C** presents a plot of the pillar force versus the bending of the microneedle for each frame of the shearing experiment. The slope corresponds to the microneedle’s spring constant

To investigate the force transmission from the apical to the basal side of an adherent cell, we conducted experiments during which we exerted well-defined shear forces to the apical side of mouse embryonic fibroblasts (MEFs) while simultaneously measuring the change in traction forces at their basal side. We employed MEFs expressing mNeonGreen (NeonG) labeled zyxin as a marker for focal adhesions. Cells were allowed to spread on a fibronectin-functionalized polyacrylamide (PAAm) gel with embedded red fluorescent marker beads so that traction forces could be derived from recording the displacement of the marker beads. A microneedle was installed into a micromanipulator such that the tip of the microneedle was parallel to the cell substrate. Moving the microneedle with a computer-controlled micromanipulator results in the application of a shear force to the apical cell surface. The spring constant of this microneedle was calibrated by shearing polydimethylsiloxane (PDMS) pillars prior to the cell experiments. To do so, first the Young’s modulus of the PDMS sample was measured with an AFM-based indentation method (Huth et al. 2019) (see Supplementary Information). Then, the calibration of the microneedle was carried out by moving a microneedle against a PDMS pillar (Fig. 1) and measuring the associated PDMS pillar and microneedle bending. In A and B, representative phase contrast images of the shearing of a PDMS pillar with a microneedle show the bending of pillar and microneedle due to shear forces. Knowing the geometry as well as the Young’s modulus of the pillar, the shear force is calculated from the pillar bending (Schoen et al. 2010). C shows a plot of the shear force versus the microneedle bending for each frame of the experiment. The slope of a linear fit to this curve corresponds to the microneedle’s spring constant.

**Fig. 2:**
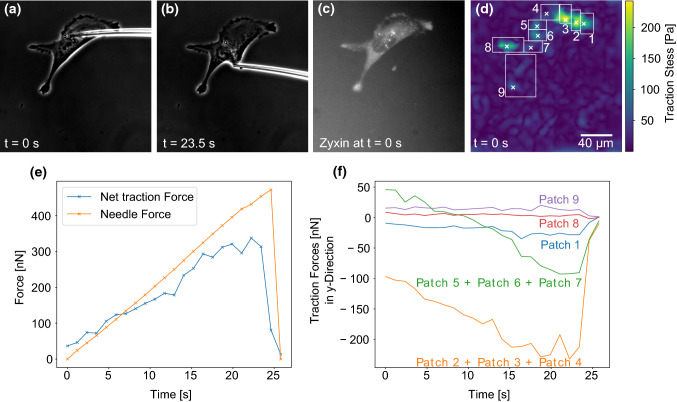
A microneedle is inserted into a fibroblast, which expresses fluorescently labeled zyxin and adheres to a TFM substrate. Subsequently, the needle is moved into the y-direction at a constant speed and exerts shear forces to the cell until it is detached. **A**, **B** Exemplary phase contrast images taken during cell shearing. Both the cell and the needle bending are monitored. The bending of the needle is used to calculate the shear force. **C** The cell’s zyxin distribution prior to the shearing process is recorded via fluorescence microscopy. **D** Traction force map of the cell with adhesion search areas delimited by white rectangles and mean patch locations marked by crosses. Traction forces were reconstructed for *t* = 0 s using Fourier Transform Traction Cytometry (FTTC). For the reconstruction of traction forces with the shear force monopole present (*t* > 0), we used the circular patch method. **E** The needle force and the cell’s net traction force are plotted as a function of time. **F** The traction forces in y-direction are plotted for different adhesion patches (labeled in panel **D**) to quantify how the cell loads its adhesion sites under the external shearing stimulus.

For the force transmission experiments, the microneedle was carefully inserted into the fibroblast cell directly below or above the nucleus. Subsequently, the microneedle was moved at a constant speed of 5 µm/s towards the nucleus to exert increasing shear forces to the cell until the cell detached from the underlying PAAm substrate. We decided to shear the nucleus, as other modes of exerting shear forces to the cell caused the microneedle to quickly slip away. This is in agreement with published work by Riveline (2001) and Paul et al. (2008) who have shown that nucleus shearing is the most efficient way to transmit forces to a cell with a microindenter. During the shearing process, phase contrast images of the cell and needle as well as fluorescent images of the marker beads embedded in the PAAm gel were recorded. Figure 2A, B presents exemplary phase contrast images of such an experiment. A video containing all phase contrast images as well as another video of the fluorescent marker beads are presented in the Supplementary Information. These phase contrast images were used to monitor the cell as well as to calculate the degree of needle bending for each frame. Knowing the needle’s spring constant, the needle bending is a measure for the shear force exerted to the cell. To correlate the measured traction forces with the distribution of adhesion sites, the zyxin distribution of the fibroblast prior to each experiment was recorded (Fig. 2C). This information is essential, as focal adhesions are the main site of traction force exertion (Balaban 2001; Sabass et al. 2008).

The images of the fluorescent marker beads embedded in the PAAm sample to which the cell is adhering were used as the basis for computing the traction forces that the cell exerted to the PAAm sample as a function of external shear force. As the microneedle applies external shear forces to the cell surface, cellular traction forces are no longer balanced by internal forces only, and the overall force balance has to include the force applied by the microneedle. In other words, the cell traction is not dominated by the force dipole contribution, as it is usually the case, but also includes a force monopole. Our experimental setup, therefore, requires several modifications to the force reconstruction algorithms commonly used in TFM (Style 2014; Soiné 2015). Due to the existence of a force monopole, deformation is very long-ranged and boundary effects must be considered. In Fourier space, the $k=0$ mode becomes relevant, which cannot be reconstructed with the standard Fourier Transform Traction Cytometry (FTTC) procedures due to the divergence of the Green’s function at $k=0$. Finally, TFM usually uses the inverse method which requires regularization, but this procedure tends to underestimate absolute force values, which are especially important in our context (Soiné 2015).

Due to these limitations, we avoided Fourier space methods (Butler et al. 2002; Sabass et al. 2008) and worked directly in real space. Although continuous force distributions can in principle be reconstructed with the boundary element method (BEM) (Dembo and Wang 1999; Han et al. 2015), here we make additional use of the fact that the cells used in our experiments have well-defined adhesion sites that are increasingly stressed as the cell is sheared by the microneedle. Motivated by this observation, we use a method where localized forces are distributed inside the cell contour (Schwarz 2002; Delanoë-Ayari et al. 2010; Schoen et al. 2013; Aramesh 2020). Rather than using point forces (Schwarz 2002), which also suffer from the divergence problem, we use known contact mechanics solutions for traction forces transmitted on circular areas (Johnson 1985; Huang et al. 2020), for which the divergence of the Green’s function is removed by integration over the contact region. The adhesion forces are estimated using the known deformation–force relation for a constant traction applied over a circular area. Recent studies have suggested that adhesion sites have in fact more elliptic shapes (Kim and Wirtz 2013; Schwarz 2002; Soiné 2015; Zamir 1999; Prager-Khoutorsky 2011). This does, however, not have a significant impact on the force reconstruction (see the Supplementary Information for a more thorough discussion). By summing over all adhesion sites and minimizing the deviation between experimental and estimate deformations, one arrives at the theoretical estimate for the traction force field (see the Supplementary Information for more details).

Figure 2D shows a traction force map computed with Fourier Transform Traction Cytometry (FTTC). These results are then used to determine the main sites of traction force transmission from the cell to the PAAm sample. These sites (“adhesion patches”) are marked by white crosses and numbers in the figure panel. In the patch method, we calculated the traction force vector of each adhesion patch and then determined the magnitude of the sum of all traction force vectors. This net traction force magnitude was then compared to the needle force. As traction forces without a force monopole are balanced, introducing an externally applied force must result in a change of traction forces to balance the externally applied force. Our results shown in Fig. 2E demonstrate that the net traction force and the externally applied shear force closely matched during the entire experiment, validating our approach. The fact that the shear force and net traction force do not match perfectly might have several reasons: force can be dissipated (Selhuber-Unkel 2010) or cells might resist deformation with cell specific responses. Furthermore, as several calibration steps are needed during force calculations, our results are prone to calibration errors: The needle spring constant was calibrated via shearing a PDMS pillar and the Young’s modulus of the PDMS was measured for the computation of the needle’s spring constant. Furthermore, the PAAm’s Young’s modulus needed to be determined in order to reconstruct traction forces from the bead displacement data. Both materials’ elastic properties were measured with a state-of-the-art atomic force microscopy procedure (Huth et al. 2019) naturally prone to measurement errors, which means that neither the needle force, nor the traction forces are perfectly accurate. Image analysis inaccuracies in the quantification of the needle bending and bead displacement may further contribute to the slight mismatch between the net traction force and externally applied force.

**Fig. 3:**
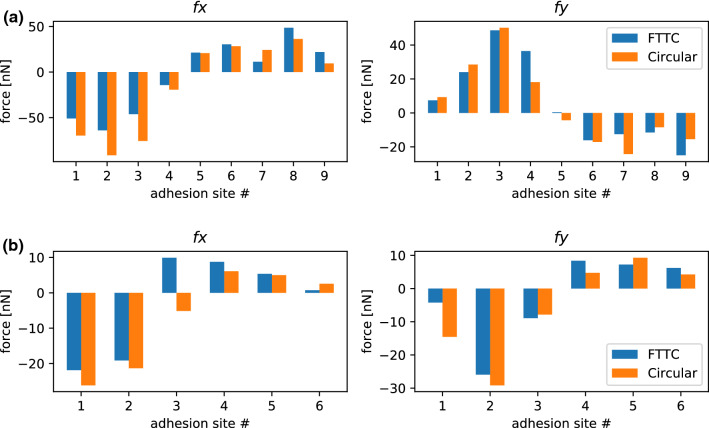
Comparison of the traction forces predicted in the absence of an external force monopole (at *t* = 0) using the circular patch method (that we employed during this study) and a regularized Fourier Transform Traction Cytometry (FTTC) (Schwarz and Soiné 2015) using generalized cross-validation (Huang 2019). **A** Profile for the cells introduced in Fig. 2. **B** Profile for the cells introduced in Fig. 5. In both cases, the agreement between the two methods is rather good

In Fig. 2F, the y-components of traction force vectors are plotted for the different adhesion patches. For better visualization, we combined some neighboring adhesion patches with similar force loading behavior. The data for each individual adhesion patch are presented in Fig. 5 of the Supplementary Information. As the needle pulled mainly in the *y*-direction, the *x*-components of the traction vectors were not influenced by the needle shear force, which is why we concentrate on discussing the y-components (a graph of the *x*-components of the traction vectors are presented in the Supplementary Information). One sees that the microneedle pulling mainly loads the adhesion patches 2, 3 and 4, and to a lesser extend also the adhesion patches 5, 6 and 7. This result had to be expected due to the position of these adhesions in the part of the cell that is tensed by the needle. On the other hand, Patches 8 and 9 are not or only slightly loaded, presumably because they are located in the part of the cell subjected to compressive forces during needle shearing. The plot shows that loading is not homogeneous and most likely is related to cytoskeletal elements (e.g., between adhesions and nucleus) not visible here. The asymmetric response of different adhesion patches can be explained by the fact that the cytoskeleton is made from semiflexible polymers, which respond differently to pulling and pushing. Pulling reduces entropy and increases stretching as well as bending energies, eventually leading to strain stiffening (Storm et al. 2005). Pushing, on the other hand, meets little resistance, because cytoskeletal filaments tend to buckle under force and the cytoplasm can flow away, thus it is difficult to locally build up compression energy like in a solid (Brangwynne 2006; Bischofs et al. 2008). It is interesting to note that also in the physiological context, cell mechanics is probed mainly in pulling, not in pushing, e.g., in epithelial monolayers, which are under large prestress (Harris 2012). Therefore, pulling is the relevant mode and much more meaningful than pushing. Thus, patches 2–7 were loaded presumably because the needle pulling forces were transmitted efficiently to these adhesion patches through the polymers of the cytoskeleton. Correspondingly, patches 8 and 9 were probably not loaded because pushing forces are not transmitted well by cytoskeletal polymers (Gardel et al. 2008). This is in agreement with earlier studies (Paul et al. 2008; Riveline 2001; Butler et al. 2002), but our results quantify the traction forces for individual focal adhesion patches under an external mechanical stimulus in an unprecedented way. Our findings also demonstrate the complexity and non-uniform distribution of intracellular force transmission as a function of load and location.

**Fig. 4:**
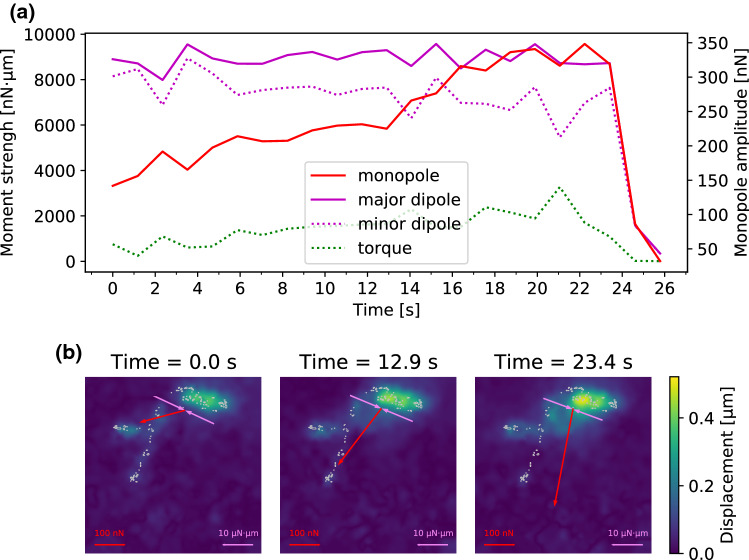
Change of force monopole and dipole moments of the cell presented in Fig. 2 in response to needle shearing. **A** presents the magnitudes of the force monopole, as well as the major and minor dipole moments and the torque as functions of time. Our results show that the contractile forces are initially distributed mostly isotropically around the contractile center. However, the force monopole created by the needle shearing increases over time while the minor dipole, which describes the contractility in the direction of the force, decreases only slightly. **B** shows the force monopole and the major dipole moment in exemplary force maps recorded during the shearing experiment. The force monopole is denoted by red arrows while the dipole moment is represented by purple arrows. The gray encircled regions represent areas where adhesions are predicted from the cell’s zyxin distribution

**Fig. 5:**
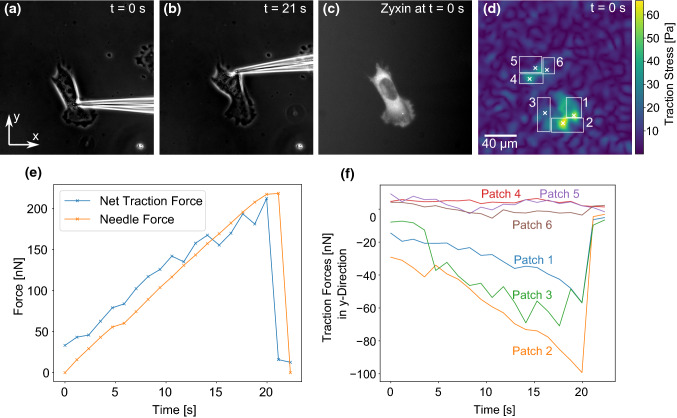
The change of traction forces as a response to microneedle shearing. **A**, **B** show phase contrast images of a cell adhering to a PAAm substrate and a microneedle exerting shear forces to the cell. The cell’s zyxin distribution prior to the shearing process is presented in **C** while **D** pictures a traction force map with cell’s adhesion patch positions marked with white crosses and numbers. The force map is calculated using FTTC at t = 0 s. In **E** The shear force exerted by the needle to the cell is compared to the magnitude of the net traction force vector. The *y*-components of the traction vectors for the adhesion patches are plotted for each moment of the experiment in **F**

The good agreement between the needle force and the net traction force predicted with our circular patch method shown in Fig. 2 E is a first and successful validation of our approach. To further validate it, we reconstructed forces at $t=0$ (when there is no force monopole) at single patches with Fourier Transform Traction Cytometry (FTTC) with $0^{\text {th}}$ order Tikhonov regularization (Schwarz and Soiné 2015), where the regularization parameter is determined by generalized cross-validation (Huang 2019). Adhesion forces were then calculated by integrating the traction stress in each search window, both for the cell analyzed in Fig. 2 and the cell analyzed in Fig. 5. As shown in Fig. 3, the agreement between the two methods is rather good in both cases.

Because an unperturbed cell to lowest order forms a force dipole, while the needle presents a force monopole, we next calculated the force moments as a function of time. Because momentum and also angular momentum is not conserved anymore due to the external pulling, one has to be careful how to define these moments (explained in Supplementary Information). Fig. 4A shows that for the cell shown in Fig. 2, the monopole increases with time, but the major dipole does not decrease as expected. The torque remains low but shows a slight upwards slope. The explanation is provided by Fig. 4B, which explicitly shows the monopole (in red) and the major dipole (in purple). Because they are oriented perpendicularly to each other, the microneedle pulling does not perturb the cellular dipole for a long time, until complete failure occurs.

**Fig. 6:**
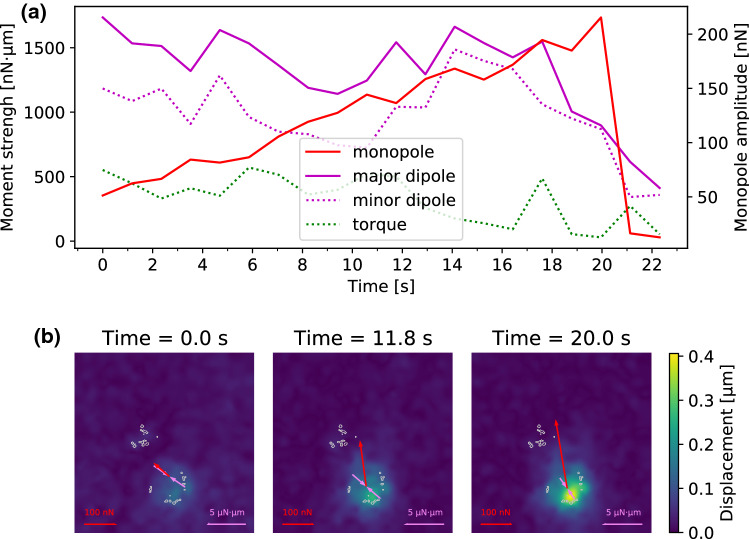
Change of force monopole and dipole moments of the cell presented in Fig. 5 in response to needle shearing. **A** presents the magnitudes of the force monopole, as well as the major and minor dipole moments and the torque as functions of time. Our results show that the force balance is initially governed by the major dipole moment. However, the force monopole created by the needle shearing increases over time and governs the force balance at high shearing forces. **B** shows the force monopole and the major dipole moment in exemplary force maps recorded during the shearing experiment. The force monopole is denoted by red arrows while the dipole moment is represented by purple arrows. The gray encircled regions represent areas where adhesions are predicted from the cell’s zyxin distribution

We now turn to an example in which monopole and dipole orientations are co-linear. For the cell presented in Fig. 5, only adhesion patches 1, 2 and 3, which—in contrast to patches 4, 5 and 6—are loaded in tension, experience an increase in their traction forces. Furthermore, the traction forces exerted through patch 2 change most strongly. This is another indication that force components perpendicular to the shear force vector are not affected by the shearing process as patch 2 lies directly below the site of shear force exertion and thus has much weaker traction forces perpendicular to the shearing direction than patches 1 and 3. Figure 5 E shows that the total traction forces exerted through the cell have the same magnitude as the needle shear force, which confirms the validity of our approach. In Fig. 6, we plot the force monopole as well as the major and minor dipole moments measured during the experiment presented in Fig. 5 as functions of time. These data demonstrate that the force balance changes from a situation that is governed by the major dipole moment to one dominated by the force monopole that is created by the needle shearing. While the adhesions in front of the needle are less exposed to the stress, the ones behind are subjected to large tensile cytoskeletal forces. Interestingly, the cellular dipole becomes more and more localized to the tensed region, indicating a strong reorganization or rearrangement also inside the cell. This is supported by the torque that experiences a downward slope indicating that the adhesive center becomes more aligned with the microneedle.

**Fig. 7:**
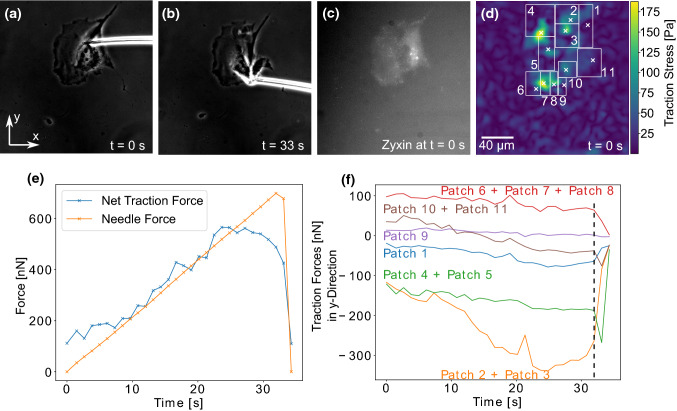
Redistribution of adhesion patch loading after a rupture event. **A**, **B** show phase contrast images of a microneedle shearing a fibroblast on a PAAm substrate. The cell’s zyxin distribution is visualized in **C**. A map of the traction forces at *t* = 0 s exerted at the cell’s adhesion patches is presented in **D**. The white crosses mark the cell’s adhesion sites. The force map is calculated using FTTC at *t* = 0 s. The sum of traction forces has roughly the same magnitude as the external shear force, as can be seen in **E**. The y-components of the traction vectors for the adhesion patches are plotted in **F**. The dashed line marks the rupture of adhesion patches 1, 2 and 3 at *t* = 32 s

The results presented in Fig. 7 show once more that not all adhesion patches are loaded with forces. Patches 1 as well as 4 and 5 were not loaded under an external shear force. Interestingly, not only patches 2 and 3, which were closest to external force application site were loaded, but also adhesion patches 10 and 11, even though they were further away from the needle than patches 1, 4 and 5. These data suggest that internal transmission of tension can be long-ranged, for example through stress fibers, as recently demonstrated by optogenetic control of cell contractility (Oakes 2017). To analyze this important aspect in detail, future work has to simultaneously image also the actin cytoskeleton. However, this is very challenging, as we also have to image the zyxin-marked focal adhesions and the fluorescent marker beads in the elastic substrates.

**Fig. 8:**
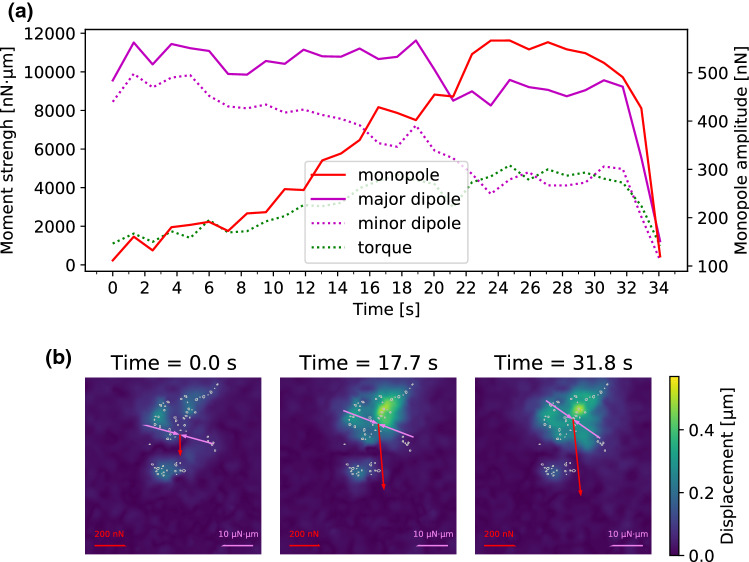
Change of force monopole and dipole moments of the cell presented in Fig. 7 in response to needle shearing. **A** Presents the magnitudes of the force monopole, as well as the major and minor dipole moments and the torque as functions of time. Our results show that the force balance is initially governed by the major dipole moment. However, the force monopole created by the needle shearing increases over time and governs the force balance at high shearing forces. **B** shows the force monopole and the major dipole moment in exemplary force maps recorded during the shearing experiment. The force monopole is denoted by red arrows while the dipole moment is represented by purple arrows. The gray encircled regions represent areas where adhesions are predicted from the cell’s zyxin distribution

It is a well-established fact that focal adhesions rupture successively under external forces (Selhuber-Unkel 2010), nonetheless our results presented in Fig. 7 quantify for the first time the redistribution of traction forces throughout the cell after the rupture of adhesion sites. When adhesion patches 1, 2 and 3 ruptured after 32 s (marked by the dashed line)—even though adhesion patch 1 had barely been loaded with force before that—the traction forces exerted through all other patches except patch 9 increased substantially. Interestingly, patches 10 and 11, which had been the only patches that were loaded strongly prior to the rupture event, were only loaded with a small amount of force upon the rupture event, while patches 4 and 5, which had been only marginally loaded, changed their traction forces much more strongly following the rupture event. In the future, one might use adhesive micropatterns to control the exact location of the adhesion patches and therefore the way individual adhesion sites are loaded by the shearing force.

In Fig. 8, we present the force monopole as well as the major and minor dipole moments measured during the experiment presented in Fig. 7 as functions of time. The behavior is similar to the one presented in Fig. 6.

Another striking aspect is the fact that the traction forces exerted through patches 2 and 3 started to slowly decrease several seconds prior to the rupturing event. We observed a similar behavior for adhesion patch 3 of the cell presented in Fig. 5. This suggests that the rupture of focal adhesions is not necessarily an instantaneous event, but that there exist rupture processes of extended duration, which we recorded using our novel analysis approach. Strikingly, the load on some focal adhesions decreased prior to rupture while in others, the traction forces increased until they ruptured. Similar differences in adhesion site behavior have been described before as slip bonds and catch bonds (Pereverzev et al. 2005), but in our experimental setting, which analyzes the behavior of intact cells, the mechanical properties of the cell and force transmission through the cytoskeleton likely play an important role, too. Our new technique, hence, enables us to reveal possible physical factors that influence dynamic changes in force loading of adhesion sites.

## Conclusion

We have introduced a novel method for determining traction forces in cells under external shear forces. The applicability of our method has been proven by shearing fibroblasts off their underlying PAAm substrates while monitoring the change in cellular traction forces at specific adhesion sites. We have shown that cells on soft substrates distribute an external shear force non-uniformly among their adhesion sites, as a function of location and load (tensile vs. compressive). Notably, we found that force transmission can be long ranged and mainly applies to adhesions that are under tensile load. This result may be due to the polymeric nature of the cytoskeletal network, which is better suited for the transmission of tensile forces. As our technique monitors the change in traction forces simultaneously to the shearing stimulation, it introduces a new quality to the recordings of rupture events to complement conventional techniques such as the single-cell force spectroscopy. Indeed, our method can be easily adapted to other force exertion methods and hence is very versatile and complementary to existing procedures. In the future, it could be combined with imaging of the cytoskeleton to achieve a more complete understanding of how force is transmitted through the cell. Moreover, adhesive micropatterns might be used to better control the positioning of the adhesion patches; with these two elements in place, we expect that our method can be used to achieve a comprehensive understanding of how force is transmitted through adherent cells.

## Materials and methods

### Cell culture

Mouse embryonic fibroblasts expressing mNeonGreen labeled zyxin were cultured at 37 $^\circ$C in 21% O$_2$, 5% CO$_2$ at a humidity of 95%. Dulbecco’s Modified Eagle Medium (DMEM; Biochrom) containing 10% fetal bovine serum (FBS; Biochrom) and 1% penicillin/streptomycin (pen strep; 10.000 $\mu g/ml$; Biochrom) served as cell culture medium. Cells were seeded on a PAAm sample by removing the cell culture medium and rinsing the cells at a confluency of about 80 % with PBS. The cells were incubated in trypsin/EDTA (0.5%/0.2% in 10xPBS; Biochrom) at 37 $^\circ$C for 1 min. Cell culture medium was added to stop the trypsination process and the cells were separated from the liquids via 5 min of centrifugation at 2412 rpm. The supernatant was replaced by fresh cell culture medium. The cells were redispersed and 100 µl of cell suspension added to a PAAm sample with another 900 µl of DMEM. The cells were allowed to spread on the PAAm sample overnight at 37 $^\circ$C prior to shearing experiments.

### Polyacrylamide preparation

A $\diameter$ 50 mm FluoroDish Cell Culture Dish (World Precision Instruments) was pretreated to promote PAAm attachment. They were cleaned three times with ethanol and double-distilled water before being incubated in sodium hydroxide (NaOH, 2.5 M) for 10 min. Subsequently, the slides were cleaned in an ultrasonic bath in double-distilled water for 10 min, rinsed with ethanol and incubated for 15 min in a mixture of 97 % ethanol (absolute), 2 % 3-(Trimethoxysilyl)propyl methacrylate (Sigma-Aldrich) and 1 % acetic acid (Sigma-Aldrich). Subsequently, they were rinsed with ethanol and dried in air. A marker bead solution was prepared by adding 100 µl fluorescent beads (1% solids, nominal $\diameter$ 50nm, Flash Red, Bangslabs, Cat. No. FSFR001) in 900 µl double-distilled water. The solution was cleaned twice by centrifuging and replacing of the supernatant with double-distilled water.

PAAm was produced by degassing a solution of 150 acrylamide µl (40%; Biorad), 90 µl bis-acrylamide (bis; 2%; Biorad), 10 µl 4-(2-hydroxyethyl)-1-piperazineethanesulfonic acid buffer (HEPES; 0.5 mM; pH = 7; Biochrom), 255 µl aqueous Acrylic acid N-hydroxysuccinimide ester solution (2 wt%, Sigma-Aldrich, CAS No 38862-24-7), 3 µl NaOH (2.5 M), and 10 µl marker bead solution in vacuum for 20 min. Subsequently, 2.5 µl ammoniumperoxodisulfate (10 wt% in aqueous solution; Sigma-Aldrich; CAS No 7727-54-0) and 0.375 µl N,N,N’,N’-Tetramethylethylenediamine (TEMED, Sigma-Aldrich, CAS No 110-18-9) were added to 260 µl of the acrylamide solution. After thoroughly mixing the solution, 10 µl were deposited into a pretreated FluoroDish and covered with a round $\diameter$ 18 µm coverslip. The sample was left to polymerize in darkness for 30 min before the coverslip was removed. The sample was soaked in double distilled water for 3 days. The water was removed and the sample was incubated in 100 µl fibronectin (aqueous 100 µg/ml solution) overnight at 6 $^\circ$C. The sample was shaken in 70% ethanol for 10 min and rinsed three times with sterile double-distilled water. Cells were added and allowed to spread overnight prior to shearing experiments. Young’s modulus of exemplary PAAm samples were measured to be $16.5 \pm 0.5$ kPa employing our priorly published procedure (Huth et al. 2019. Details are published in the Supplementary Information.

### Microneedle preparation

Microneedles were pulled from hollow borosilicate glass tubes (outer diameter 1 mm, inner diameter 0.5 mm, length 100 mm, item $\#$: B100-50-10, Sutter Instruments Co.) using a Flaming/Brown micropipette puller (Model P-97, Sutter Instruments Co.). The employed parameters were Pressure = 500, Heat = 490, Velocity = 70, Pull = 70, Time = 100. For descriptions of these parameters please refer to (Oesterle 2018). A MF-900 Microforge (NARISHIGE Group) was used to bend the needle so that the tip and base form an angle of about 45$^\circ$. Each needle was installed into a micromanipulator so that its tip is parallel to the sample surface.

### Microneedle calibration

Each time a needle was installed to the micromanipulator, its spring constant was calibrated prior to cell shearing experiments by shearing the needle against a PDMS pillar. Prior to calibration, a PDMS sample with a network of 6 µm long pillars with a radius of 2 µm was soaked in water and degassed in vacuum for 10 min to remove air bubbles. The microneedle was positioned next to a PDMS pillar. The optimum needle height was determined by lowering the microneedle in 1 µm steps and trying to shear the pillar after each step to see if the needle slips before the pillar bends. Subsequently, the needle was moved against the pillar at 2 µm/s while phase contrast images were recorded at a frame rate of 0.85 fps. At least one image of the unbent needle was acquired before the shearing process was initiated. This image served as a reference for calculation of needle bending. To eradicate errors from needle assymetry, the direction of needle movement was chosen to be the same during cell shearing experiments and during the calibration process. To calculate the spring constant, the positions of the needle tip and the PDMS pillar were tracked manually in phase contrast images with imageJ. The respective positions in the reference frame were subtracted to compute the distances the tip had moved and the PDMS pillar had bent. For each frame, the time stamps of the phase contrast images were employed to calculate how much time has passed since the needle movement had started. This duration was multiplied with the speed of needle movement to compute the distance the needle had moved. The microneedle bending was calculated as the difference between micromanipulator distance and needle tip distance. The force necessary to bend a PDMS pillar was calculated as published by (Schoen et al. 2010). Young’s modulus of the PDMS sample had been measured to be $801.5 \pm 32.9$  kPa employing our previously published procedure (Huth et al. 2019). The pillar force was plotted versus the needle bending and a linear fit is employed to calculate the slope of the resulting curve that corresponds to needles spring constant. We present an exemplary calibration experiment as well as details on the determination of the PDMS sample’s Young’s modulus in the Supplementary Information.

### Shearing process and shear force calculation

A fluorescence image of the zyxin distribution of a well-spread fibroblast was recorded. The calibrated microneedle was inserted into this cell directly above or below the nucleus and a phase contrast image of cell and needle was recorded. Subsequently, the needle was moved horizontally at 5 µm/s against the nucleus. During the shearing process, phase contrast images of cell and needle as well as fluorescent images of the marker beads embedded in the underlying PAAm substrate were recorded alternately at a frame rate of 0.85 fps. After cell detachment, an additional pair of fluorescent microscopy and phase contrast images was recorded. This last fluorescent image pair recorded the bead position of the PAAm sample without any influence of traction forces and served as reference image for traction force calculations.

Images were recorded using an inverted microscope (Z1 Observer, Zeiss) equipped with a CMOS Camera (Hamamatsu ORCA Flash 4.0) and a 40x objective with phase contrast (Zeiss EC Plan-Neofluar 40x/0.75 Ph2 M27). Both, the phase contrast images and the fluorescence images were recorded using the RFP filtercube (necessary to image the fluorescent marker beads) to minimize the time between the measurement of shear force and traction forces. The microneedle was handled using a Eppendorf InjectMan NI2 micromanipulator. For each frame, the bending of the needle was calculated as described in the calibration section above. The shear force was computed by multiplying the needle bending with the needle’s spring constant calibrated prior to each experiment.

### Calculation of traction forces

Traction forces were calculated using a home-written algorithm that employs the established deformation-force relation for a constant traction applied over a circular area. A single adhesion was assumend per area. The location of each adhesion center within each area was determined for each frame using the local maxima approach. The adhesion radius was found by inspecting the surrounding peaks. A common radius was determined for each adhesion, which was then used for all frames. A detailed description can be found in the Supplementary Information. The substrate deformation field was obtained from fluorescent bead images using PIV (Westerweel 1997; Taylor et al. 2010). A windows size of 64 pixels and a 50% window overlap were used. Spurious vectors were removed using a minimal signal-to-noise ratio in the correlation function of 1.5 and a threshold of 2.0 for the normalized median test. The reference image was taken after cell detachment.

## Supplementary Information

Below is the link to the electronic supplementary material.

Supplementary file1 (AVI 1631 KB)

Supplementary file2 (AVI 6578 KB)

Supplementary file3 (AVI 1568 KB)

Supplementary file4 (AVI 2759 KB)

Supplementary file5 (PDF 537 KB)
