# Supplementary material for: Quantifying force transmission through fibroblasts: changes of traction forces under external shearing
Source: Eur Biophys J. 2021 Oct 28;51(2):157–69. doi: 10.1007/s00249-021-01576-8 (PMC8964583; doi:10.1007/s00249-021-01576-8)
Supplement: Supplementary file 5 — Supplementary file5 (PDF 537 KB) [file 249_2021_1576_MOESM5_ESM.pdf]

Quantifying Force Transmission through Fibroblasts: Changes of Traction Forces under External Shearing - Supplementary Information

Steven Huth, Johannes Blumberg, Dimitri Probst,
Jan Lammerding, Ulrich S. Schwarz and Christine Selhuber-Unkel

Reconstruction of traction forces

In our analysis, we assume that focal adhesions, where force is transmitted to the substrate, have a circular shape. For the far field, it is not essential whether the traction forces within an adhesion are distributed evenly or vary over this area, for example decaying towards the rim. In any case, the far field would result in the Green's function of the elastic halfspace, which represents a point force in the middle of the adhesion [1]. For the near field, the solutions looks slightly different. Therefore the main difference between different assumptions would be the reconstruction of the forces in the middle of the focal adhesions. To compare the effect of different assumptions, we initially consider two types of circular adhesive patterns: the constant traction force patterns and radial decreasing Hertz-like traction force pattern.

The analytical solution for the surface deformation created by a tangential traction force $\mathbf{F} = (F_x, F_y)^T$ distributed equally over a circular area with radius a at the surface of a sufficiently thick, substrate in the linear, isotropic elastic regime has recently been calculated in the context of traction force microscopy [2]. Employing polar coordinates $\mathbf{r} = r(\cos\theta, \sin\theta)$ centered around the middle of the circular adhesion, the surface traction profile is given by:

$$\boldsymbol{\tau}(r,\theta) = \begin{cases} \frac{\mathbf{F}}{\pi a^2} & r < a \\ 0 & r \geq a \end{cases} \tag{1}$$

The corresponding deformation field is given by

$$u_x(r,\theta) = \frac{1+\nu}{\pi^2 a E}\left[(1-\nu)N_1(r,\theta) + \nu N_2(r,\theta))F_x - \nu N_3(r,\theta)F_y\right] \tag{2}$$

$$u_y(r,\theta) = \frac{1+\nu}{\pi^2 a E}\left[-\nu N_3(r,\theta)F_x + (1-\nu)N_1(r,\theta) + \nu N_4(r,\theta))F_y\right] \tag{3}$$

Here the E describes the substrate stiffness (Young's modulus) and ν the Poisson ratio. The functions N_1 to N_4 have the following form in the inner region where

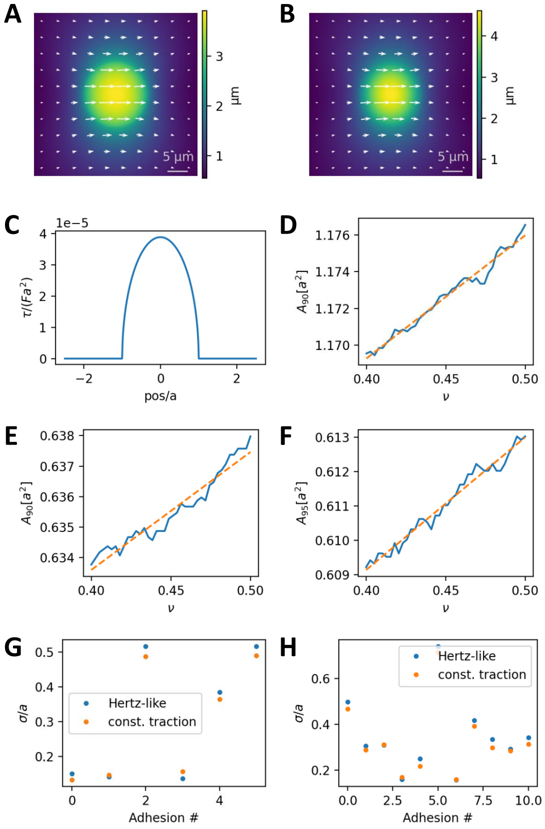

Figure 1: **A** Plot of the surface deformation created by a constant-traction profile with $a = 8\,\mu\text{m}$, $F = \pi a^2 \cdot 1.5\,\text{kPa}$, $E = 6.9\,\text{kPa}$, $\nu = 0.5$. **B** Plot of the surface deformation created by a Hertz-like profile, same parameters as in A **C** cross-section for the Hertz like profile. **D** Numerical relation between between A_{90} and ν for the constant-traction profile **E** Same relation for the Hertz-like profile. **F** Numerical results relation between between A_{95} and ν for the constant-traction profile **G** Comparison of the relative standard derivation in the definition of areas for the cell presented in Fig. 5 of our main manuscript. **H** Same for the cell presented in Fig. 2 of our main manuscript.

$r < a$ and $\xi_1 = r^2/a^2$:

$$N_1 = 4E_0(\xi_1) \tag{4}$$

$$N_2 = \frac{4\cos(2\theta)\left((r^2+a^2)E_0(\xi_1) + (r^2-a^2)K_0(\xi_1)\right)}{3r^2} + 4\sin^2\theta E_0(\xi_1) \tag{5}$$

$$N_3 = \frac{2\sin(2\theta)\left((r^2-2a^2)E_0(\xi_1) + (r^2-a^2)K_0(\xi_1)\right)}{3r^2} \tag{6}$$

$$N_4 = 4\cos^2\theta E_0(\xi_1) - \frac{4\cos(2\theta)\left((r^2+a^2)E_0(\xi_1) + (r^2-a^2)K_0(\xi_1)\right)}{3r^2} . \tag{7}$$

Here, E_0 and K_0 describe the complete elliptic integral of the first and second kind, respectively. For the outer region where $r > a$ and $\xi_2)a^2/r^2$ we have

$$N_1 = \frac{4\left(r^2E_0(\xi_2) + (a^2-r^2)K_0(\xi_2)\right)}{ar} \tag{8}$$

$$N_2 = \frac{\left(6r^2 - 2(r^2-2a^2)\cos(2\theta)\right)E_0(\xi_2) + 2(r^2-a^2)(\cos(2\theta)-3)K_0(\xi_2)}{3ar} \tag{9}$$

$$N_3 = \frac{2\sin(2\theta)\left((r^2-2a^2)E_0(\xi_2) + (a^2-r^2)K_0(\xi_2)\right)}{3ar} \tag{10}$$

$$N_4 = \frac{\left(6r^2 + 2(r^2-2a^2)\cos(2\theta)\right)E_0(\xi_2) - 2(r^2-R^2)(\cos(2\theta)+3)K_0(\xi_2)}{3ar} . \tag{11}$$

The shape of the deformation field is shown in Fig. 1.

In the limit $r \to 0$ we find that

$$\mathbf{F} = \frac{\pi a E}{(1+\nu)(2-\nu)}\mathbf{u}(0) . \tag{12}$$

This is the relation between overall force and displacement in the middle of the focal adhesion. For the Green's function, this displacement would diverge, because it only describes the far field.

The surface deformation created by a tangential traction force distributed in a Hertz-like manner over a circular area is known from contact mechanics [3]. Employing Cartesian coordinates centered around the center of the circular adhesion, and the abbreviation $r = \sqrt{x^2+y^2}$ the surface traction profile is given by:

$$\boldsymbol{\tau}(x,y) = \begin{cases} \frac{3\mathbf{F}}{2\pi a^3}\sqrt{a^2-r^2} & r < a \\ 0 & r \geq a \end{cases} \tag{13}$$

A linear cross-section of this deformation profile is shown in Fig. 1. The corresponding deformation field is given by:

$$u_x(x,y) = \frac{3(1+\nu)}{8Ea^3}((W_1+W_2)F_x + W_3F_y) \tag{14}$$

$$u_y(x,y) = \frac{3(1+\nu)}{8Ea^3}(W_3F_x + (W_1+W_4)F_y) \tag{15}$$

The functions W_1 to W_4 have the following form in the inner region where $r < a$:

$$W_1 = \frac{1}{4} 4(2-\nu)a^2 \tag{16}$$
$$W_2 = -\frac{1}{4}((4-3\nu)x^2 + (4-\nu)y^2) \tag{17}$$
$$W_3 = \frac{1}{4} 2\nu xy \tag{18}$$
$$W_4 = -\frac{1}{4}((4-3\nu)y^2 + (4-\nu)x^2)\,. \tag{19}$$

For the outer region where $r > a$ and $\xi_2 = a^2/r^2$ we have:

$$W_1 = \frac{2-\nu}{\pi}\left((2a^2 - r^2)\arcsin\frac{a}{r} + ar\sqrt{1-\frac{a^2}{r^2}}\right) \tag{20}$$
$$W_2 = \frac{\nu}{2\pi}\left(r^2\arcsin\frac{a}{r} + (2a^2 - r^2)\frac{a}{r}\sqrt{1-\frac{a^2}{r^2}}\right)\frac{x^2-y^2}{r^2} \tag{21}$$
$$W_3 = \frac{1}{\pi}\left(r^2\arcsin\frac{a}{r} + (2a^2 - r^2)\frac{a}{r}\sqrt{1-\frac{a^2}{r^2}}\right)xy \tag{22}$$
$$W_4 = \frac{\nu}{2\pi}\left(r^2\arcsin\frac{a}{r} + (2a^2 - r^2)\frac{a}{r}\sqrt{1-\frac{a^2}{r^2}}\right)\frac{y^2-x^2}{r^2}\,. \tag{23}$$

The shape of this deformation is also shown in Fig. 1.

In the limit $r \to 0$, we find, that:

$$\mathbf{F} = \frac{8aE}{3(1+\nu)(2-\nu)}\mathbf{u}(0)\;. \tag{24}$$

Compared with Eq. (12), we see that both scale linear in Young's modulus and patch size. Also the contribution related to the Poisson ratio is equivalent. They differ only in the constant prefactor.

In general, the deformation fields in both cases share many similarities. In both cases the absolute value of the deformation field $u_{abs} = \sqrt{u_x^2 + u_y^2}$ takes its maximal value at the center of the deformation. In addition the isolines of the u_{abs} field enclose simple connected regions always containing the center of the coordinate system. We define A_h to be the area where $u_{abs}(x,y) > h u_{abs}(0,0)$. While the deformation field $\mathbf{u}$ is dependent on five parameters E, ν, a, F_x and F_y, only two of them a and ν will affect A_h. Because of the way we can choose our unit scale, it can be easily seen, that a contributes quadratically ($A_h \propto a^2$). A numerical analysis (Fig. 1) reveals that the relation to ν can be estimated using a linear function. Therefore, the expression $A_h = (y_h + m_h\nu)a^2$ describes the relationship between A_h, a and ν. The constants y_h and m_h can be determined numerically by simulating the situation for an arbitrary choice of the five parameters mentioned above. An estimation of the total force $\mathbf{F}$ of

an Hertz-like or constant-traction contact based on the deformation field can be found using Eq. (12) or (24), by finding the deformation at the a center of the contact. The contact radius a can be calculated from the isoline-enclosed area A_h for some value h.

The overall algorithm to determine forces $\mathbf{F}_i$ within each adhesion now contains the following steps done individually for each adhesion search area i:

1. Interpolate the deformation field onto a regular spaced square grid for each time step.

2. Calculate the absolute value $u_a bs$ of the deformation field for each time step.

3. Locate the center of the adhesion by making use of the fact that $u_a bs$ should reach its maximum in this location for each time step.

4. A common issue in the above estimation is the fact that the adhesion in adjacent search areas might cause the center of the current adhesion not to correspond to the global maximum of $u_a bs$ within its search area, in which case the largest value for $u_a bs$ can be found right next to the adhesion search area boundary. In these cases, we rely on an interpolation from the other time-steps to select the presumed location for the area estimate.

5. Now that we have determined the center of the adhesion for each time step and the radius a, we can determine the deformation $\mathbf{u}$ in the adhesion center.

6. Calculate the area A_h within the adhesion search area where $u_a bs$ lies within $1 - h = 5\,\%$, $10\,\%$, $20\,\%$, $30\,\%$ of its maximal value for each time step.

7. For each time step and each threshold value an estimate for the adhesion radius a can now be determined using the above mentioned area formula $a = \sqrt{A_h/(y_h + m_h \nu)}$ using the predetermined values for y_h and m_h In general, all of this estimates should yield a similar value, as the radius of the adhesion is expected not to change during the procedure.

8. The final estimate for the adhesion radius can now be found by finding the mean of the estimates determined in the previous step. We explicitly emit those time steps form the calculation, where we had to use the interpolation from the other time-steps in step 4, as the estimates in these cases are particularly unreliable.

9. Now that we have determined the deformation $\mathbf{u}$ in the center of each adhesion for each time step and the radius a, we can determine the corresponding force $\mathbf{F}_i$ using Eq. (12) or (24).

In order to determine whether adhesion sites can be better described by Hertz-like or the constant-traction profiles, we compare the statistic variance

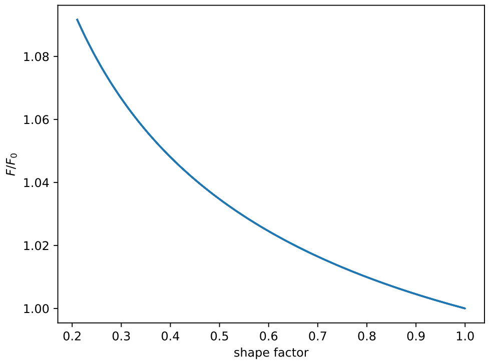

Figure 2: Ratio between the predicted force for an elliptical contact and a circular contract of equal contact area F/F_0 for $\nu = 0.5$ using Eq. (26). The horizontal axes describes the shape factor given as defined by [4].

between the radius estimates derived from different search area estimates in step 7. As can see (Fig 1. G and H) both approaches give a similarly consistent image in this regard. As the constant-force approach gives a slightly better estimate, we chose the constant traction estimate for our experimental analysis.

These two approaches assume a circular shape of the adhesion area, while many recent studies have suggested a more elliptic shape [4]. In case of a Hertz like contact, the surface traction profile can be modeled, if we assume that both the direction of the force, as well as one of the semi-axes are parallel to the x-axes:

$$\boldsymbol{\tau}(x,y) = \begin{cases} \frac{3F_x\mathbf{e}_x}{2\pi ab}\sqrt{1-\frac{x^2}{a^2}-\frac{x^2}{b^2}} & \frac{x^2}{a^2}+\frac{y^2}{b^2} < 1 \\ 0 & \frac{x^2}{a^2}+\frac{y^2}{b^2} \geq 1 \end{cases} \tag{25}$$

The deformation field for force central displacement relation at the center is given by [5]:

$$\mathbf{F} = \frac{8\sqrt{ab}E}{3N(\nu,a/b)(2-\nu)(1+\nu)}\mathbf{u}(0) \ . \tag{26}$$

The function $N(\nu, a/b)$ is given by

$$N\left(\nu, \frac{a}{b}\right) = \begin{cases} \frac{4}{\pi(2-\nu)}\sqrt{\frac{a}{b}}\left(K_0(m_a) - \nu\frac{K_0(m_a)-E_0(m_a)}{m_a}\right) & a < b \\ 1 & a = b \\ \frac{4}{\pi(2-\nu)}\sqrt{\frac{b}{a}}\left((1-\nu)K_0(m_b) + \nu\frac{K_0(m_b)-E_0(m_b)}{m_b}\right) & a > b \end{cases} \tag{27}$$

with the definition $m_a = 1-a^2/b^2$ and $m_b = 1-b^2/a^2$. Its inverse $1/N$ describes the ratio between the force predicted using Eq. (26) and the one predicted using Eq. (24) for a circular adhesion of equal area. In Fig. 2 $1/N$ is plotted against the

shape factor $\frac{\pi^2}{4}\frac{b}{a}\frac{1}{E_0(m_a)^2}$. Assuming a shape factor of 0.5 as observed by [4], we see that the force increase is only around 4% which is likely below the accuracy of the prediction.

Calculation of force moments

The force monopole is the net directed force on the substrate and defined by

$$\mathbf{F} = \int \boldsymbol{\tau} d^2x \ . \tag{28}$$

Knowing the adhesion patches, this means that we can find the total force monopole vector by simply summing up the force contributions of all adhesions:

$$\mathbf{F} = \sum_k \mathbf{F}_k \ . \tag{29}$$

Due to momentum conservation, the force monopole should be equivalent to the force transmitted by the cantilever into the cell.

We also define the first order moment matrix $\mathbf{M}$ as [6]

$$M_{ij} = \int x_i \tau_j d^2x \ . \tag{30}$$

The coordinate frame for this integral is chosen with respect to the center of force of the system, which is given by

$$\boldsymbol{x}_{\mathrm{CF}} = \left(\int |\boldsymbol{\tau}| d^2x \right)^{-1} \int |\boldsymbol{\tau}| \boldsymbol{x} d^2x \tag{31}$$

which can be calculated in any coordinate frame.

Inserting the traction profile for patches (Eq. 13 or Eq. 1) yields:

$$\boldsymbol{x}_{\mathrm{CF}} = \left(\sum_k |\mathbf{F}_k| d^2x \right)^{-1} |\mathbf{F}_k| \boldsymbol{x}_k d^2x \ , \tag{32}$$

$$M_{ij} = \sum_k (\boldsymbol{x}_k)_i (\mathbf{F}_k)_j d^2x \ . \tag{33}$$

The diagonal components of the moment matrix describe the contractility of the system. The two off-diagonal components corresponds to a torque relative to the center of force [6]. We define the contractile momentum by

$$\mu = M_{11} + M_{22}. \tag{34}$$

This describes the net ability to dilate or contract the cell. The net torque is defined by

$$\mathcal{M} = M_{12} - M_{21}. \tag{35}$$

Both μ and $\mathcal{M}$ are independent of the orientation of the coordinate axes and are independent of each other.

Without needle pulling, angular momentum conservation dictates that the net torque is zero and the moment matrix symmetric. In this case, one can find

an orientation of the axis such that **M** is diagonal and the eigenvalues can be used to find the directed and isotropic contractile moment of the system. To also consider the case of needle pulling, we define a slightly modified version of the moment matrix, where we removed the torque contribution:

$$M_{ij}^{\parallel} = \int x_i x_j \frac{\boldsymbol{\tau} \cdot \boldsymbol{x}}{\boldsymbol{x}^2} d^2x \ . \tag{36}$$

If we again insert the definition of the patches, we obtain:

$$M_{ij}^{\parallel} = \sum_k (\boldsymbol{x}_k)_i (\boldsymbol{x}_k)_j \frac{\mathbf{F}_k \cdot \boldsymbol{x}_k}{\boldsymbol{x}_k^2} \ . \tag{37}$$

This matrix is symmetric and thus an orthogonal eigendecomposition can be found. The two eigenvalues describe the dipole moments and the eigenvector corresponding to the major dipole describes the main contractile axis. This can be proven by comparing the trace of $\boldsymbol{M}^{\parallel}$ to the contractile momentum μ, which both yield the same value.

Young's modulus of PDMS-pillars used for microneedle calibration

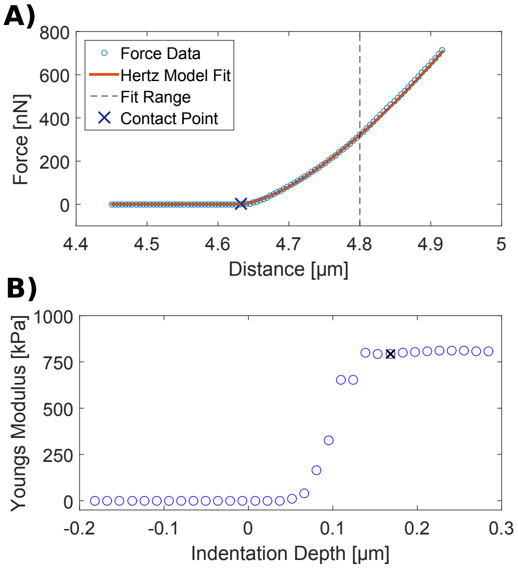

Figure 3: The Young's modulus of the PDMS pillars employed for the calibration of the microneedle was measured using an AFM indentation based method published by Huth et al. [7]. A silica bead with 21 µm diameter was glued to an AFM cantilever and was subsequently used to indent the PDMS sample with a setpoint force of 700 nN at 1 µm/s. **A** shows an exemplary indentation curve with a Hertz model fit. Our method employs an algorithm that calculates the Young's modulus for different indentation depths. The resulting curve is presented in **B**. The Young's modulus is determined by finding a plateau in this curve and finding the value with lowest fitting residuals. This value is marked in **B** with a black cross. The sample was indented 16 times at each of 32 different positions resulting in a mean value of 801.49 +/- 32.91 kPa.

Young's modulus of the polyacrylamide sample

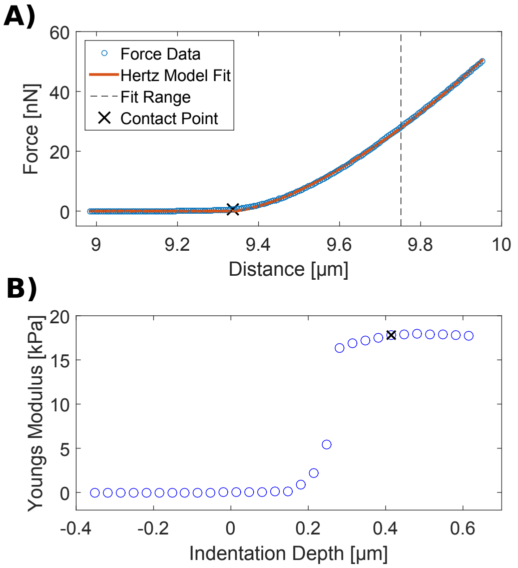

Figure 4: The Young's modulus of a polyacrylamide (PAAm) sample that serves as a traction force microscopy substrate needs to be known in order to calculate traction forces from the displacement of the beads embedded in the sample. We employed the same method to measure the Young's modulus of our PAAm samples as for the PDMS pillars. Indentation curves were collected at a cantilever speed of 1 µm/s and a setpoint force of 50 nN. **A** shows an exemplary indentation curve with a Hertz model fit, while **B** shows the resulting Young's modulus versus indentation depth. The sample was indented 16 times at each of 30 different positions resulting in a mean value of 16.49 +/- 0.55 kPa.

Traction forces in perpendicular direction

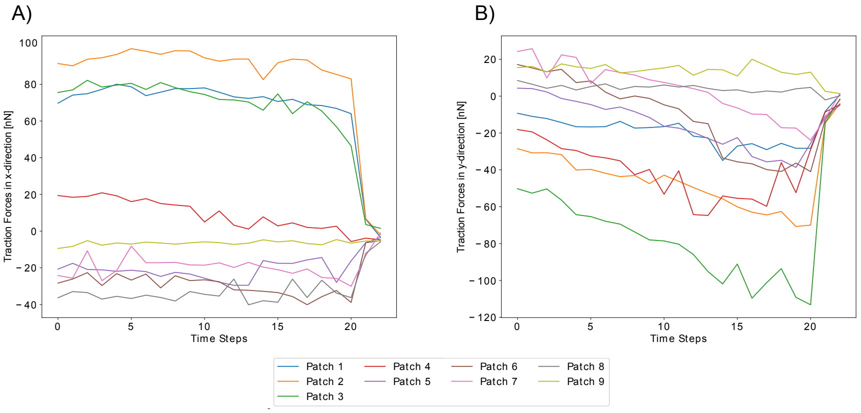

Figure 5: Traction force data from the cell presented in Fig. 2 of our main manuscript. **A** shows the x-components of the traction force vectors. It is very clear the these are not influenced by the shearing in y-direction, which is why we focus our discussion on traction forces in y direction. **B** shows the y-components of the traction forces for each adhesion patch. We decided to combine some neighbouring patches with similar behavior for better visualization. We combined patches 2,3 and 4 as well as 5,6 and 7.

References

[1] U.S. Schwarz, N.Q. Balaban, D. Riveline, A. Bershadsky, B. Geiger, and S.A. Safran. Calculation of forces at focal adhesions from elastic substrate data: The effect of localized force and the need for regularization. *Biophysical Journal*, 83(3):1380–1394, 2002.

[2] Yunfei Huang, Christoph Schell, Tobias B Huber, Ahmet Nihat Şimşek, Nils Hersch, Rudolf Merkel, Gerhard Gompper, and Benedikt Sabass. Traction force microscopy with optimized regularization and automated Bayesian parameter selection for comparing cells. *Scientific Reports*, 9(1):539, 2019.

[3] Kenneth L. Johnson. *Contact Mechanics*. Cambridge University Press, 1985.

[4] Dong-Hwee Kim and Denis Wirtz. Focal adhesion size uniquely predicts cell migration. *The FASEB Journal*, 27(4):1351–1361, 2013.

[5] P. J. Vermeulen and K. L. Johnson. Contact of nonspherical elastic bodies transmitting tangential forces. *Journal of Applied Mechanics*, 31(2):338–340, June 1964.

[6] James P. Butler, Iva Marija Tolić-Nørrelykke, Ben Fabry, and Jeffrey J. Fredberg. Traction fields, moments, and strain energy that cells exert on their surroundings. *American Journal of Physiology-Cell Physiology*, 282(3):C595–C605, March 2002.

[7] Steven Huth, Sandra Sindt, and Christine Selhuber-Unkel. Automated analysis of soft hydrogel microindentation: Impact of various indentation parameters on the measurement of Young's modulus. *PLoS ONE*, 14(8):e0220281, 2019.
